# High CD8^+^ and absence of Foxp3^+^ T lymphocytes infiltration in gallbladder tumors correlate with prolonged patients survival

**DOI:** 10.1186/s12885-018-4147-6

**Published:** 2018-03-02

**Authors:** Paula Fluxá, Daniel Rojas-Sepúlveda, María Alejandra Gleisner, Andrés Tittarelli, Pablo Villegas, Loreto Tapia, María Teresa Rivera, Mercedes Natalia López, Felipe Catán, Mario Uribe, Flavio Salazar-Onfray

**Affiliations:** 1 Disciplinary Program of Immunology, Institute of Biomedical Sciences, Faculty of Medicine, Universidad de Chile, 8380453 Santiago, Chile; 2 Millennium Institute on Immunology and Immunotherapy, Faculty of Medicine, Universidad de Chile, 8380453 Santiago, Chile; 3 Unidad de Anatomía Patológica, Hospital del Salvador, 7500922 Santiago, Chile; 4 Departamento de Cirugía Oriente, Faculty of Medicine, Universidad de Chile, 8380453 Santiago, Chile

**Keywords:** Gallbladder cancer, Tumor infiltrating lymphocytes, Patient survival, CD8^+^ T cells, Foxp3^+^ T cells

## Abstract

**Background:**

Gallbladder cancer (GBC), although infrequent in industrialized countries, has high incidence rates in certain world regions, being a leading cause of death among elderly Chilean women. Surgery is the only effective treatment, and a five-year survival rate of advanced-stage patients is less than 10%. Hence, exploring immunotherapy is relevant, although GBC immunogenicity is poorly understood. This study examined the relationship between the host immune response and GBC patient survival based on the presence of tumor-infiltrating lymphocytes at different disease stages.

**Methods:**

Tumor tissues from 80 GBC patients were analyzed by immunohistochemistry for the presence of CD3^+^, CD4^+^, CD8^+^, and Foxp3^+^ T cell populations, and the results were associated with clinical stage and patient survival.

**Results:**

The majority of tumor samples showed CD3^+^ T cell infiltration, which correlated with better prognosis, particularly in advanced disease stages. CD8^+^, but not CD4^+^, T cell infiltration correlated with improved survival, particularly in advanced disease stages. Interestingly, a < 1 CD4^+^/CD8^+^ T cell ratio was related with increased survival. Additionally, the presence of Foxp3^+^ T cells correlated with decreased patient survival, whereas a ≤ 1 Foxp3^+^/CD8^+^ T cell ratio was associated with improved patient survival.

**Conclusions:**

Depending on the disease stage, the presence of CD8^+^ and absence of Foxp3^+^ T cell populations in tumor tissues correlated with improved GBC patient survival, and thus represent potential markers for prognosis and management of advanced disease, and supports testing of immunotherapy.

**Electronic supplementary material:**

The online version of this article (10.1186/s12885-018-4147-6) contains supplementary material, which is available to authorized users.

## Background

Gallbladder cancer (GBC) is the most common cancer of the biliary tree. Although GBC is infrequent in developed countries [1], in South America this tumor constitutes a major health problem [1, 2]. In particular, Chile has the highest incidence and mortality rates of GBC in the world, being this cancer the leading cause of neoplastic disease-related deaths in women (12.8 cases per 100,000) [1, 3, 4]. The underlying causes for the high risk of GBC in Chile are unclear, but several important risk factors likely contribute, including chronic inflammation caused by gallstones, high obesity rates, and genetic susceptibility in women of indigenous *Mapuche* ancestry, in which the incidence rise up to 27.3 cases per 100,000 [1, 3–5].

The most effective GBC treatment is surgical removal of the primary tumor and areas of local extension. Unfortunately, less than 10% of the patients have resectable tumors, and nearly 50% of them present metastasis at the time of diagnosis [6]. In fact, even after surgery, most GBC-patients progress to a metastatic stage, stressing the need for novel adjuvant therapies, such as immunotherapy. Recently, we reported that dendritic cell (DC)-immunization could improve long-term survival in melanoma and prostate cancer patients [7–9]. A way to determine the potential usefulness of DC-based immunotherapy in GBC patients is to explore the immunogenicity of GBC tumors by measuring the impact of T cell subpopulation infiltration at tumor sites and correlate it with overall patient survival.

The presence of tumor-infiltrating lymphocytes (TILs) in cancer tissues is indicative of an active host immune response against cancer cells. CD4^+^ and CD8^+^ T cells are the main components of tumor-specific, cellular adaptive immunity. Indeed, several studies have shown that a high presence of tumor-infiltrating CD8^+^ T cells is associated with favorable prognosis in colorectal, ovarian, breast, pancreatic, and biliary tract cancers (BTC) [10–14]. However, this is not a general principle that can be applied to all kinds of tumors [15, 16].

CD4^+^ T cells are also essential for regulating immune responses, a role exercised mainly through the secretion of different cytokines [17, 18]. During the activation of T cell receptors in a local cytokine milieu, CD4^+^ T cells can differentiate into several lineages of effector T cells or regulatory T cells (Tregs), as defined by cytokine expression and cell function patterns. Tregs can inhibit effector T cell functions in physiological and illness states through cell-cell contact or by the secretion of regulatory cytokines, such as IL-10 and TGF-β [19]. Foxp3 is a transcription factor and a Treg-specific marker that regulates Treg development [20]. Considering that Tregs within the tumor microenvironment might significantly suppress local antitumor immune responses [21], increased presence of Foxp3^+^ T cells in peripheral blood or tumor tissues have been associated with negative prognoses for various cancers [22–24]. In BTC, sub-populations of infiltrating immune cells have so far only been studied in parts, using relatively small and heterogeneous patient cohorts, and without a defined disease stage analysis [25, 26].

The purpose of this study was to evaluate the prognostic significance of various TIL subtypes, including CD4^+^, CD8^+^, and Foxp3^+^ T cells, and to analyze the influence of density and distribution of tumor-infiltrating immune cell populations at different stages of the disease, in a homogeneous cohort of Chilean GBC patients with a long term follow up. Our results suggest that the balance between CD8^+^ and Foxp3^+^ cells in the tumor microenvironment acts as a predictor for GBC patient survival, a situation that allows speculating that boosting natural antitumor immune responses through immunotherapy could represent a possible adjuvant treatment for GBC patients.

## Methods

### Patients

Between 1996 and 2004, resected tumor samples were obtained from 64 female and 16 male GBC patients (80 patients total) treated at the Hospital del Salvador (Santiago, Chile). The stage of GBC was assigned according to the TNM Classification of Malignant Tumors (7th edition, 2010). Patients were selected on the basis of suitable paraffin-embedded tissue, as well as the availability of a complete clinical-pathology and follow-up data. All patients included in the study died due to GBC. The cause of death was verified in the database of the national civil registry.

Patients were classified by disease stage as either early stage (stages 0, I; pTNM Is, Ia and Ib) or advanced stage (stages II, III and IV). The early GBC stage patients were defined by the presence of adenocarcinoma confined to the tunica muscularis (Tis, T1a, or T1b) according the AJCC/UICC, TNM Classification 2010. The study was performed in agreement with the Helsinki Declaration and approved by the Bioethical Committee for Human Research of the Faculty of Medicine, University of Chile (ID number: 078–2008). All patients signed a letter of informed consent at the time of surgery (For patient characteristics please refer to Table 1).

**Table 1:** Demographic and clinical characteristics of patients

	Age range	Indian American Ethnicity^a^	pTNM	Stage	Histological differentiation	Survival (Months)	Adjuvant Treatment	CD3	CD4	CD8	FoxP3
1	30/79	No	TisN0M0	0	In Situ	60	No	5	7	8	0
2		ND	TisN0M0	0	In Situ	48	No	36	24	16	8
3		No	TisN0M0	0	In Situ	60	No	34	23	22	4
4		Yes	TisN0M0	0	In Situ	60	No	20	16	1	5
17		No	TisN0M0	0	In Situ	60	No	46	39	18	2
6		ND	T1bN0M0	I	Moderate	18	No	38	27	12	2
7		No	T1bN0M0	I	Well	60	No	2	2	2	0
8		No	T1aN0M0	I	Well	60	No	14	21	46	2
9		ND	T1bN0M0	I	Well	3	No	22	17	20	1
10		ND	T1aN0M0	I	Well	60	No	32	24	19	2
11		No	T1bN0M0	I	Well	60	No	36	16	32	0
12		No	T1bN0M0	I	Poorly	60	No	28	22	19	0
13		No	T1aN0M0	I	Well	60	No	14	18	10	0
14		No	T1aN0M0	I	Moderate	60	No	20	18	23	0
15		No	T1bN0M0	I	Moderate	60	No	15	14	22	0
16		No	T1bN0M0	I	Well	29	CT + RT	16	12	10	8
18		Yes	T1bN0M0	I	Moderate	60	No	28	14	27	0
19		No	T1bN0M0	I	Well	60	CT + RT	42	26	26	17
20		Yes	T1bN0M0	I	Moderate	0	No	15	8	0	9
21	38/85	No	T2N0M0	II	Poorly	0	No	12	1	14	3
22		No	T2N0M0	II	Moderate	60	ND	26	15	24	0
23		No	T2N0M0	II	Well	60	ND	21	15	38	5
24		N.D	T2N0M0	II	Moderate	13	ND	13	11	2	2
25		ND	T2N0M0	II	Moderate	22	ND	18	14	11	6
26		No	T2N0M0	II	Moderate	6	ND	24	31	2	1
27		No	T2N0M0	II	Moderate	60	CT + RT	22	23	17	3
28		No	T2N0M0	II	Moderate	0	No	19	18	14	7
29		No	T2N0M0	II	Poorly	7	ND	13	13	3	0
30		Yes	T3N0M0	IIIA	Poorly	3	No	18	7	8	0
31		No	T3N0M0	IIIA	Moderate	60	No	34	4	25	0
32		No	T3N0M0	IIIA	Well	19	ND	23	17	34	1
33		No	T3N0M0	IIIA	Poorly	4	No	80	70	38	5
34		No	T3N0M0	IIIA	Moderate	8	No	21	22	8	1
35		No	T3N0M0	IIIA	Well	60	ND	24	25	7	0
36		No	T3N0M0	IIIA	Moderate	11	No	27	11	22	1
37		No	T3N0M0	IIIA	Moderate	3	No	45	20	6	1
38		No	T3N0M0	IIIA	Moderate	2	No	16	20	5	9
39		ND	T3N0M0	IIIA	Moderate	60	ND	36	10	31	20
40		ND	T3N0M0	IIIA	Poorly	6	ND	ND	32	ND	ND
41		No	T3N0M0	IIIA	Poorly	4	No	18	23	21	5
42		ND	T3N0M0	IIIA	Moderate	4	ND	22	10	16	13
43		No	T3N0M0	IIIA	Poorly	60	CT	12	13	26	7
44		No	T3N0M0	IIIA	Moderate	0	No	10	12	8	2
45		No	T3N0M0	IIIA	Well	0	No	16	14	3	8
46		No	T3N0M0	IIIA	Poorly	5	ND	39	30	32	4
47		No	T3N0M0	IIIA	Moderate	3	No	15	5	4	3
48		ND	T3N0M0	IIIA	Moderate	4	ND	10	11	18	0
49		ND	T3N0M0	IIIA	Moderate	6	ND	25	25	19	10
50		Yes	T3N0M0	IIIA	Poorly	11	ND	26	15	7	0
51		No	T3N0M0	IIIA	Moderate	9	ND	22	19	9	3
52		No	T3N0M0	IIIA	Moderate	1	No	5	6	8	0
53		No	T3N0M0	IIIA	Poorly	2	No	11	9	12	6
54		No	T3N0M0	IIIA	Moderate	5	ND	37	17	30	0
55		ND	T3N0M0	IIIA	Moderate	4	ND	12	12	21	7
56		No	T3N0M0	IIIA	Poorly	1	No	37	42	0	1
57		No	T3N0M0	IIIA	Moderate	2	No	14	10	0	6
58		No	T3N0M0	IIIA	Moderate	4	No	10	7	0	1
59		No	T2N1M0	IIIB	Moderate	60	ND	37	15	54	11
60		No	T2N1M0	IIIB	Poorly	60	CT + RT	17	6	25	1
61		ND	T3N1M0	IIIB	Poorly	5	ND	28	20	17	4
62		ND	T3N1M0	IIIB	Moderate	16	ND	15	14	19	1
63		No	T3N1M0	IIIB	Moderate	5	No	32	19	27	15
64		No	T2N1M0	IIIB	Well	60	CT + RT	40	31	36	2
65		No	T2N1M0	IIIB	Moderate	7	ND	21	19	10	1
66		ND	T3N1M0	IIIB	Poorly	5	No	20	17	26	26
67		Yes	T2N1M0	IIIB	Poorly	60	ND	30	18	30	0
68		No	T2N1M0	IIIB	Moderate	10	CT + RT	19	14	17	7
69		ND	T3N1M0	IIIB	Poorly	11	CT + RT	28	31	32	10
70		No	T2N1M0	IIIB	Moderate	60	CT + RT	23	21	33	5
71		No	T2N1M0	IIIB	Moderate	51	ND	23	12	13	1
72		ND	T3N1M0	IIIB	Poorly	2	No	33	8	16	0
73		No	T3N1M0	IIIB	Moderate	12	ND	11	14	17	10
74		No	T2N1M0	IIIB	Poorly	60	CT + RT	23	13	10	2
75		No	T4N1M0	IVA	Poorly	3	No	45	40	12	3
76		No	T4N0M0	IVA	Moderate	9	ND	20	26	8	ND
77		No	T3N2M0	IVB	Moderate	34	CT + RT	13	16	19	0
78		No	T3N2M0	IVB	Moderate	14	ND	27	18	0	1
79		No	T3N2M0	IVB	Poorly	9	ND	51	44	34	5

^a^*Mapuche* ethnical origin was assessed by correlation with patient parents last, family, or middle name

*ND* No Data, *CT* Chemotherapy, *RT* Radiotherapy. White = early stage of disease. Light grey = Late stage of disease

### Immunohistochemistry

Tissue specimens were fixed in 10% formalin and embedded in paraffin wax. Representative blocks, which included the greatest dimensions of the tumor sections, were selected for immunohistochemistry analysis using the streptavidin-biotin-peroxidase method. Paraffin blocks were dissected into 3 μm thick slides, mounted on silanized slides, and subsequently dewaxed and rehydrated using xylene and graded ethanol washes, followed by washing with phosphate-buffered saline (pH 7.4). The heat-induced epitope retrieval protocol, with 20 min heating and 30 min cooling, was used to retrieve antigens. The slides were placed on a retrieval solution (Tris-EDTA, pH 9.0) or citrate buffer (pH 6.0) depending on each antibody. Endogenous peroxidase activity was blocked with 0.3% hydrogen peroxide for 30 min followed by Tris-buffered saline washing. A protein block solution was applied for 45 min to stop non-specific antibody binding. The following primary antibodies were used according to the manufacturers’ instructions: monoclonal rabbit IgG anti-CD3, clone SP7 (1:250 dilution; Thermo Scientific); monoclonal rabbit IgG anti-CD4, clone SP35 (1:25 dilution; BioSB); monoclonal mouse IgG anti-CD8, clone C8/144B (1:50 dilution; DAKO); and monoclonal mouse IgG anti-Foxp3, clone 236A/E7 (1:50 dilution; Abcam).

The slides were incubated with the primary antibodies in moist chambers for 45 min at room temperature. Then, the slides were washed with three changes of Tris-buffered saline before incubation with labeled polymer horseradish peroxidase secondary antibodies for an additional 45 min at room temperature (Universal Secondary Antibody, ABC Vectastain Kit Elite PK6200, Vector Laboratories). Slides were subsequently incubated with the ABC solution for 30 min (ABC Vectastain Kit Elite PK6200, Vector Laboratories), washed with three changes of Tris-buffered saline, incubated with DAB (3,3′-diaminobenzidine tetrahydrochloride)-Chromogen solution (DAKO) according to manufacturer’s instructions, and washed with deionized water. Background staining was performed with Mayer’s haematoxylin solution. Finally, the sections were dehydrated through ascending alcohols and xylene and then mounted. Negative control slides omitting the primary antibody were included in all batches. Sections from tonsil tissue were used as positive controls for CD3^+^, CD4^+^, and CD8^+^ cell detections.

Because of limited access to material, one patient sample was not analyzed for CD8^+^ and CD4^+^ T cell infiltration, as well as two patients samples were excluded for FoxP3^+^ T cell infiltration analysis.

### Quantification methods

Two pathologists independently scored positive for stained TILs. CD3^+^, CD4^+^, CD8^+^, and Foxp3^+^. TILs were counted using an optical microscope (40X, Olympus Bx40F-3) in five randomly selected high power fields. The cell count average was calculated, and these values were used as the final cells/field assigned to each patient.

Sturges’ Rule was used to establish categories, which were compared with one another to obtain the cut-off values for defining high and low infiltration levels. The cut-off values for each cell subtype were as follows: 23 cells/field for CD3^+^, 22 cells/field for CD8^+^, and 20 cells/field for CD4^+^ cells. Foxp3^+^ cell infiltration was analyzed as positive or negative since the different categories showed no differences. Furthermore, since the intraepithelial and peritumoral infiltration levels showed no differences in relation to patient survival, both compartments were analyzed together.

### Statistical analysis

Statistical analyses were performed using GraphPad Prism (version 5.0, GraphPad Software Incorporated). Comparisons between groups were examined by one-way analysis of variance. Overall survival was calculated according to the Kaplan-Meier method. The log-rank test was used to compare survival rates between patient subgroups based on the status of either high or low immune cell infiltration. Cox’s proportional hazards model was used for univariate analysis of cell infiltration and overall survival, with analysis performed using the Stata statistical software (version 12.1, StataCorp). Probability (*p*) values < 0.05 were considered significant in all analyses.

## Results

### Patient characteristics and infiltrating T cell numbers

The cause of death for all patients was GBC, according to databases of the national civil registry. All cases of cancer evaluated in this study were diagnosed as adenocarcinoma of the gallbladder (*n* = 79). Only one patient was excluded from the statistic study because the histopathological analysis of the biopsy diagnosed a carcinoid of the gallbladder. For all remaining 79 patients with GBC, pathological data was acquired on TNM classification, differentiation, vessel involvement, lymphatic involvement, perineural involvement, and stage grouping. Follow-up and clinical data were also obtained from all patients. Information regarding basic clinical-pathology characteristics, treatment and overall patient survival time is summarized in Table 1, and Additional file 1: Figure S1.

**Fig. 1:**
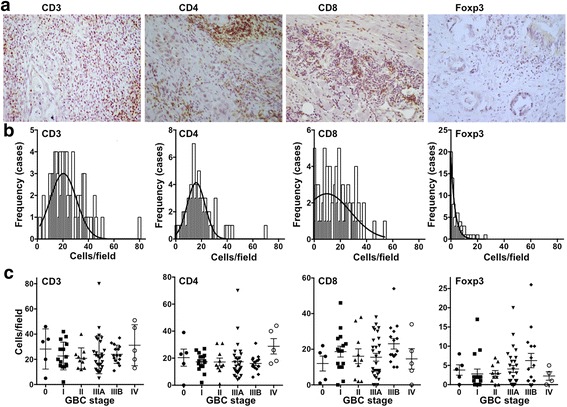
Immune cell infiltration in gallbladder cancer. **a** Representative photomicrographs of immunohistochemical staining T cells (CD3^+^, CD4^+^, CD8^+^, and Foxp3^+^) within GBC tissues (scale bar, 40 μm). **b** Quantified distribution of immune cell infiltration by photomicrograph field in GBC tissues. The frequency is displayed as the number of cases. The superimposed black lines show the fitted Gaussian distribution. Gaussian distribution was only found on CD4^+^ T cells (goodness of fit: R^2^ = 0.796). **c** Quantified distribution of immune cell infiltration in GBC tissues according to GBC stage. Data represent the mean ± SD

Representative immunohistochemistry images for CD3^+^, CD4^+^, CD8^+^, and Foxp3^+^ T cell infiltrations in GBC samples are provided in Fig. 1a. All patient samples (*n* = 79) showed immune cell infiltration in the peritumoral and intratumoral compartments. When the immune cells infiltrating each compartment were independently quantified, no statistical associations with overall GBC patient survival were found (data not shown). Distributional analysis by photomicrograph field for all immune cell infiltrate quantities revealed that only the number of CD4^+^ TILs evidenced Gaussian distribution (Fig. 1b). Additionally, total counts of CD3^+^, CD4^+^, CD8^+^, and Foxp3^+^ T cells were similar in all GBC samples, regardless of the disease stage (Fig. 1c).

### CD3^+^ T cell infiltration correlates with increased survival of advanced stage gallbladder cancer patients

**Fig. 2:**
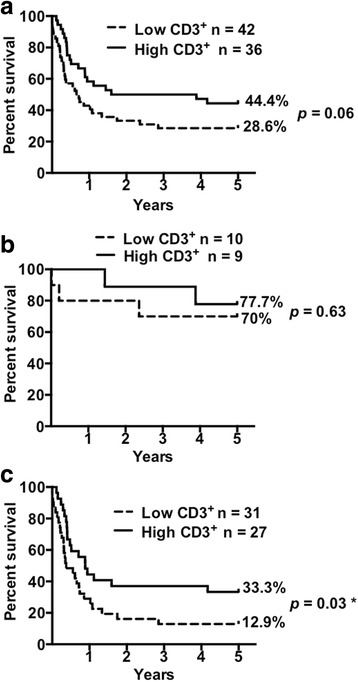
Increased CD3^+^ T cell infiltration in gallbladder cancer tissues correlates with improved late stage patient survival. Kaplan-Meier survival curves for patients with high and low CD3^+^ T cells infiltration in primary tumors based on Sturges’ Rule: (**a**) All stages; (**b**) Early stages (0 and I); and (**c**) Advanced stages (II, IIIA, IIIB, and IV). High CD3^+^ T cell infiltration was significantly associated with prolonged survival in advanced stage patients (log-rank *p* = 0.03). The survival proportions of patients after GBC diagnosis in a 5-year term are shown to the right of the curves

The GBC tumor tissues were grouped into two categories, according to the degree of CD3^+^, CD4^+^, or CD8^+^ T cell infiltration and either high or low infiltration rate. Out of 79 patients with positive CD3^+^ T cell tumor infiltration, 42 (53.8%) were classified as low CD3^+^ infiltration rate and 36 (46.2%) as high. The median survival time of GBC patients with low and high CD3^+^ infiltration rates were 8.37 and 33.3 months (*p* = 0.06), respectively (Fig. 2a). Early (stage 0 and I) and advanced stage (II, III and IV) patients were separately grouped and analyzed. There was no significant difference in median survival time between low and high CD3^+^ infiltration (*p* = 0.63) in the early stage patients (Fig. 2b). However, higher infiltration by CD3^+^ T cells was correlated with better median survival time (10.6 months vs. 4.6 months, respectively; *p* = 0.02; HR = 1.96; CI = 1.054–3.445) in advanced stage patients (Fig. 2c).

### High CD8^+^ T cell infiltration correlates with increased survival of gallbladder cancer patients

**Fig. 3:**
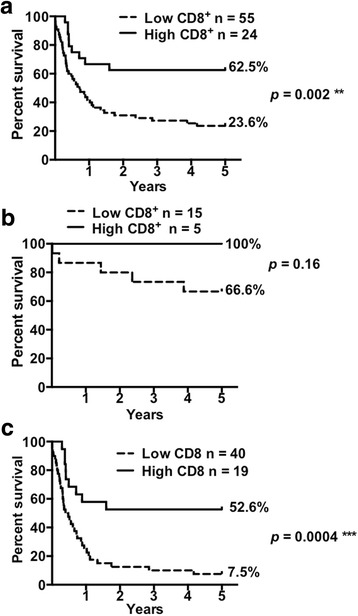
Increased CD8^+^ T cell infiltration in gallbladder cancer tissues correlates with improved patient survival. Kaplan-Meier survival curves for patients with high and low CD8^+^ T cell infiltration in primary tumors based on Sturges’ Rule: (**a**) All stages; (**b**) Early stages (0 and I); and (**c**) Advanced stages (II, IIIA, IIIB, and IV). High CD8^+^ T cell infiltration was significantly associated with prolonged survival particularly in advanced stage patients (log-rank *p* = 0.0004). The survival proportions of patients after GBC diagnosis in a 5-year term are shown to the right of the curves

Similarly, 78 patient samples were grouped by high or low CD8^+^ T cell infiltration. Patients with higher CD8^+^ T cell infiltration had greater median survival times than patients with low CD8^+^ T cell infiltration, undefined (62.5% of patients still survive) vs. 8.7 months, respectively (*p* = 0.002; HR = 2.44; CI = 1.38–4.3) (Fig. 3a). When CD8^+^ T cell infiltration was assessed within early and advanced patient groups, high CD8^+^ T cell infiltration was associated with better prognosis than low CD8^+^ T cell infiltration for the early stage group. None of the early stage GBC patients from the high CD8^+^ T cell infiltration group died during the study, whereas 33.4% of the patients with low CD8^+^ T cell infiltration died during the follow-up period (Fig. 3b). Remarkably, high CD8^+^ T cell infiltration in advanced stage patients was correlated with a greater median survival time compared to low CD8^+^ T cell infiltration, undefined (52.6% of patients survive) vs. 5.2 months, respectively (*p* = 0.0004; HR = 2.89; CI = 1.6–5.22) (Fig. 3c).

### CD4^+^ T cell infiltration was not associated with the overall survival of gallbladder cancer patients

In 78 analyzed samples, CD4^+^ T cells were found in both peri and intratumoral compartments with no relationship between distribution and stage of the disease. Additionally, when early GBC and advanced stage patients were analyzed separately, no differences in survival time were observed between high and low CD4^+^ T cell infiltration groups (Additional file 1: Figure S2).

### Foxp3^+^ T cell infiltration was rare, but associated with reduced survival of gallbladder cancer patients

**Fig. 4:**
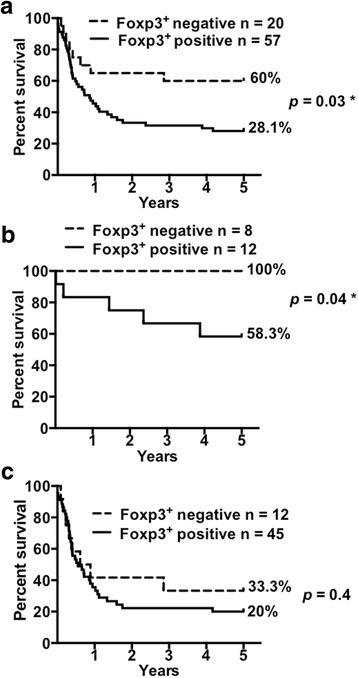
Increased Foxp3^+^ T cell infiltration in gallbladder cancer tissues correlates with poor patient survival. Kaplan-Meier survival curves for patients with positive or negative Foxp3^+^ T cell infiltration in primary tumors for (**a**) All stages; (**b**) Early stages (0 and I); and (**c**) Advanced stages (II, IIIA, IIIB, and IV). Negative Foxp3^+^ T cell infiltration was significantly associated with prolonged survival in all stages and early stage patients (log-rank *p* = 0.03 and 0.04, respectively). The survival proportions of patients after GBC diagnosis in a 5-year term are shown to the right of the curves

Assessed GBC tumor samples (*n* = 77) were poorly infiltrated by Foxp3^+^ T cells (Fig. 1b and c). Specifically, 20 samples were negative for Foxp3^+^ T cells and 57 were positive. Patients with negative Foxp3^+^ T cell infiltration had a better survival outcome than patients with positive infiltration (Fig. 4a). Indeed, while patients from the positive Foxp3^+^ T cell infiltration group showed a median survival of 10.4 months, 60% of patients with negative Foxp3^+^ T cell infiltration survived the 5-year follow-up period (*p* = 0.03; HR = 0.5; CI = 0.27–0.9) (Fig. 4a). The same association was observed when early stage patients were analyzed separately from those in the advanced stage. All early stage GBC patients with negative Foxp3^+^ T cell infiltration survived the 5-year follow-up period whereas only 58.3% of early stage patients with positive infiltration of Foxp3^+^ T cells survived (*p* = 0.04; HR = 0.16; CI = 0.02–0.95) (Fig. 4b). On the other hand, the absence of Foxp3^+^ T cells was not associated with better prognosis in advanced stage patients (Fig. 4c).

### Lower CD4^+^/CD8^+^ and Foxp3^+^/CD8^+^ T cell ratios in gallbladder cancer tissues correlates with improved patient survival

**Fig. 5:**
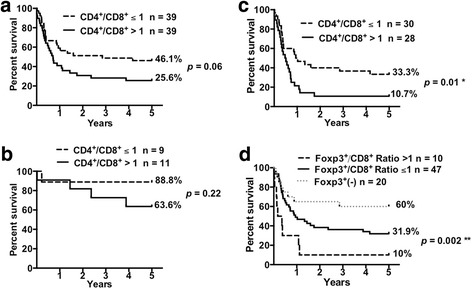
Lower CD4^+^/CD8^+^ and Foxp3^+^/CD8^+^ T cell ratios in gallbladder cancer tissues correlates with improved patient survival. Kaplan-Meier survival curves for patients by (**a**-**c**) CD4^+^/CD8^+^ T cell ratio or (**d**) Foxp3^+^/CD8^+^ T cell ratio in primary tumors for (**a**, **d**) All stages; (**b**) Early stages (0 and I); and (**c**) Advanced stages (II, IIIA, IIIB, and IV). A ≤ 1 CD4^+^/CD8^+^ T cell ratio was significantly associated with prolonged survival in advanced stage patients (log-rank *p* = 0.01). A ≤ 1 Foxp3^+^/CD8^+^ T cell ratio was significantly associated with prolonged survival in all stages patients (log-rank *p* = 0.002). The survival proportions of patients after GBC diagnosis in a 5-year term are shown to the right of the curves

Typically, variations in the proportion of different tumor-infiltrating immune cells are more accurate than absolute numbers for estimating the immune microenvironment [27, 28]. Therefore, CD4^+^/CD8^+^ T cell ratios were used to study influence of cell infiltration on the GBC patient survival. No statistical differences were found in overall survival when comparing patients with a ≤ 1 ratio to those with a > 1 ratio (*p* = 0.06; Fig. 5a). A similar situation was found when early stage patients were separately analyzed (*p* = 0.22; Fig. 5b). However, the analysis of advanced stage patients showed that a ≤ 1 CD4^+^/CD8^+^ T cell ratio correlated with improved patient median survival (11.8 months vs. 5.9 months, *p* = 0.01) (Fig. 5c.)

Similarly, an analysis of Foxp3^+^/CD8^+^ T cell ratio was performed to assess its influence on GBC patient survival. Kaplan-Meier survival analyses revealed that a > 1 Foxp3^+^/CD8^+^ T cell ratio was associated with a significant shorter median survival than patients with a ≤ 1 Foxp3^+^/CD8^+^ T cell ratio (3.2 vs. 11.5 months, respectively; *p* = 0.002) (Fig. 5d).

### CD8^+^ T cell infiltration is a prognostic predictor for survival of gallbladder cancer patients

Cox’s proportional hazards model was used to analyze if the examined parameters could act as prognostic predictors for GBC patients. Only CD8^+^ T cell infiltration was identified by Cox univariate regression as a significant prognostic biomarker (*p* = 0.003; HR = 0.24; CI = 0.09–0.62) (Additional file 1: Table S1). The multiparametric analysis of T cell infiltration respect to patient’s demographic characteristics, histopathological findings, or clinic pathological features did not show significant correlations (Additional file 1: Table S2).

## Discussion

Tumor-infiltrating immune cells are representative of the tumor immune microenvironment and constitute an accepted manifestation of the host immune response against cancer. T cells are functionally heterogeneous and consist of various immune cell subgroups. Therefore, T cells can play dual roles, potentially either controlling or promoting malignancies. Generally, while CD8^+^ T cell tumor infiltration is associated with favorable prognosis, a high prevalence of Foxp3^+^ Tregs within the tumor microenvironment indicates a malignant phenotype and adverse outcome for different kinds of cancer [10, 11, 24]. The immune system involvement in the pathogenesis and control of GBC has only been partially studied. The present work is one of the first to evaluate associations between the number of different TIL subtypes and the overall survival in a cohort of South American GBC patients at distinct disease stages.

Likewise, a possible relationship between tumor-infiltrating immune cells and GBC prognosis was previously suggested by Nakakubo et al. [29] in a pioneering study in which 45 tumor samples from GBC patients and 65 benign gallbladder tissues were examined. These authors reported an increased quantity of CD4^+^, CD8^+^ and DCs in GBC samples, and their observations significantly correlated with prolonged patient survival. In turn, a report on 375 BTC patients, including 69 GBC samples, indicated that GBC patients with intraepithelial tumor-infiltrating CD4^+^ and CD8^+^ T cells showed significantly longer overall survival [14]. However, no correlation was made between disease stage and infiltration by regulatory T cells in these studies. In a more recent study, Oguro et al. [30] analyzed 211 GBC samples and found that a lower density of tumor-infiltrating CD8^+^ cells and higher ratios between Foxp3^+^/CD4^+^, B and T lymphocyte attenuator/CD8^+^, and casitas-B-lineage lymphoma protein-b/CD8^+^ were significantly associated with shorter overall survival in GBC patients. Nevertheless, the balance between pro-inflammatory CD8^+^ tumor-infiltrating immune cell subpopulations and Foxp3^+^ T cells, and their impact at different disease stages on GBC patient survival has not yet been established.

Among our obtained results, a greater infiltration of CD8^+^ T cells in cancer tissue was associated with a favorable prognostic biomarker particularly for advanced stage GBC patients. Additionally, the presence of Foxp3+ T cells correlated with poor survival outcome for early stage patients, suggesting that these cells may be regulatory T lymphocytes and may play a role in controlling the immune response towards GBC during the disease onset. However, although Foxp3 expression is generally considered a useful marker for Tregs, its transient expression in other T cells subpopulations make that a role for Foxp3^+^ cells lacking Treg function, although improbable, may not be discarded.

Moreover, our results also indicate that CD4^+^ T cell infiltration is not associated with clinical outcomes in our GBC patient cohort. Although it is generally accepted that CD4^+^ T cells may contribute to suppression of tumor growth via T_H_1 cytokine production [18], a low CD4^+^/CD8^+^ ratio has also been associated with a better prognosis for colorectal cancer patients [31]. In that sense, our results showed that lower CD4^+^/CD8^+^ and Foxp3^+^/CD8^+^ ratios correlated with a better prognosis for all GBC patients, especially those in the advanced stages. This observation suggests that the presence of CD4^+^ T cells is insufficient to contain tumor growth in a poor CD8^+^ T cells environment, and that Foxp3+ T cells (probably Tregs) may adversely affect the ability of CD8^+^ T cells to attack GBC tumors. According to protocols available at the time of surgery, only 10 patients included in this study received adjuvant treatment (chemotherapy, radiotherapy), as shown in Table 1. However, considering that samples were obtained from surgical specimens before any treatment, the degree of immune cell infiltration found in the samples did not differ from the rest of the patients. In terms of survival, the statistical analyzes did not show any differences between the groups regarding the use of adjuvant therapies (data not shown).

Few groups worldwide have researched the influence of tumor-infiltrating immune cells on GBC prognosis. Out of a total 5 studies, two were made with Japanese patients [29, 30], two with German patients [14, 32], and one with Chinese patients [33]. Chile has the highest GBC incidence and mortality rate among women worldwide [1, 34]. The most important risk factor in Chile is high gallstone prevalence and other inflammatory associated conditions caused by bacterial infections, which leads to chronic inflammation [35]. This condition, present in all cases reported here, has not been described in any other studies and may contribute to some differences detected in infiltrating T cell subpopulations. The recurrent cycles of gallbladder epithelium damage and repair enable a chronic inflammatory environment that promotes progressive morphological impairment associated with carcinogenesis [35], and probably with an early immune cell infiltration as well.

In turn, a second inflammation-independent tumorigenesis process associated with anomalous pancreatobiliary duct junction is more frequent in Japan and China [36]. A carcinogenesis process induced by inflammation may have a different early immune response than an inflammatory-independent tumorigenesis process, explaining some differences in our results compared to other previous studies. Additionally, GBC in Chile has been associated to the *Mapuche* ethnic group [1]. Although, we failed to detect any correlation between GBC cohort and ethnicity, it is known that a great majority of the Chilean population bears more than 60% of Native American genes [37].

Indeed, our results demonstrated that all tumors of Chilean patients were infiltrated by CD3^+^ T cells, while only 30–50% of the Japanese and German samples showed this kind of infiltration [14, 30]. The discrepancy may be explained by the difference of origins and characteristics of patient cohorts, although differences in used criteria for positivity cannot be discarded. Furthermore, Nakakubo et al. [29] and Goeppert et al. [14] found that an increased quantity of total or intraepithelial CD4^+^ T cells correlates with prolonged GBC patient survival. In contrast, the present work indicates that CD4^+^ T cell infiltration, which is present in the majority of the samples, does not influence the survival of GBC patients. Differences in the GBC carcinogenesis pathways can lead to diverse anti-tumor immune responses, determining the nature of immune cell infiltration. Ultimately, these variances could impact GBC progression and overall patient survival.

## Conclusions

In conclusion, the results of this study strongly indicate that a natural host CD8^+^ T cell-mediated immune response against GBC increases patient survival. This finding could be useful in clinical decisions related to management and treatment of advanced GBC patients. Also, it encourages the design and development of adjuvant immunotherapeutic approaches against GBC. One of the most important findings of this study is that disease progression does not imply a loss of tumor immunogenicity, however, the kind of response and cell subtypes activated in tumor environment may define the response effect. In fact, immunological aspects observed in situ, particularly the presence of CD8^+^ T cells or Foxp3^+^ T cells, together with other histopathological characteristics, may permit identifying the group of patients that must be handled with a more aggressive strategy in accordance with the expected prognosis. In the future, this may facilitate the selection of candidates for immunotherapy approaches.

Some immunotherapy trials against GBC use peptide-based vaccines or peptide-loaded DCs [38, 39]. These strategies have shown modest clinical improvements, likely due to induced tolerance by a dominant single tumor peptide or the selection of antigen-negative tumor cells. In contrast, a DC vaccine using autologous tumor cell lysates combined with adoptive T cell immunotherapy in patients with intrahepatic cholangiocarcinoma reported improved progression-free and overall survival [40]. Autologous tumor cell lysates have also been tested as antigen providers for DCs in melanoma that induced a strong and more extensive immunological response against tumors [41]. A useful and promising alternative is the preparation of allogeneic cancer cell lysates that have demonstrated to provide a standardized applicable source of tumor-specific antigens, also in patients with non-resectable tumors [42, 43]. Particularly, allogeneic tumor cell lysates obtained from heat shocked conditioned tumor cells contain elevated levels of danger signals (like released HMGB1 and translocated calreticulin), which improve DC maturation and tumor antigen cross-presentation [8, 44]. In fact, clinical benefits reported by our group in the use of autologous DCs loaded with allogeneic conditioned tumor cell lysates in melanoma [7, 8] and prostate cancer [9], encouraged us to explore the use of allogeneic conditioned GBC lysates-loaded DCs in future clinical trials. Finally, considering that Chilean GBC patients are infiltrated by T cells, immune checkpoint therapies such as blocking of PD-1 or CTLA4, along or in combination with DC vaccines could be a reasonable strategy for the treatment of GBC.

## Additional file

Additional file 1: **Table S1.** Survival prognostic factors in Gallbladder cancer patients. Cox’s proportion. **Table S2.** CD3^+^, CD8^+^ and Foxp3^+^ T cell infiltration related to patient’s demographic characteristics. **Figure S1.** Overall survival of gallbladder cancer patients. Kaplan-Meier survival curves for GBC patients stratified according to the disease stage. **Figure S2.** CD4^+^ T cell infiltration in gallbladder cancer tissues does not correlates with patient survival. (DOC 264 kb)

## Abbreviations

BTC: Biliary tract cancer
CD: Cluster of differentiation
CI: Confidence interval
CTLA4: Cytotoxic T-lymphocyte antigen 4
DC: Dendritic cells
Foxp3: Forkhead box P3
GBC: GBC
HMGB1: High mobility group box-1
HR: Hazard ratio
IgG: Immunoglobulin G
IL: Interleukin
MHC: Major histocompatibility complex
PD1: Programmed cell death protein-1
TGF: Transforming growth factor
TIL: Tumor-infiltrating lymphocytes
Treg: Regulatory T cells
