# Supplementary material for: High CD8+ and absence of Foxp3+ T lymphocytes infiltration in gallbladder tumors correlate with prolonged patients survival
Source: BMC Cancer. 2018 Mar 2;18:243. doi: 10.1186/s12885-018-4147-6 (PMC5833069; doi:10.1186/s12885-018-4147-6)
Supplement: Supplementary file 1 — Table S1. Survival prognostic factors in Gallbladder cancer patients. Cox’s proportion. Table S2. CD3+, CD8+ and Foxp3+ T cell infiltration related to patient’s demographic characteristics. Figure S1. Overall survival of gallbladder cancer patients. Kaplan-Meier survival curves for GBC patients stratified according to the disease stage. Figure S2. CD4+ T cell infiltration in gallbladder cancer tissues does not correlates with patient survival. (DOC 264 kb) [file 12885_2018_4147_MOESM1_ESM.doc]

**Supplementary Material**

**Supplementary Table 1: Survival prognostic factors in Gallbladder cancer patients. Cox’s proportion.**

|  |  |  |  | HR (CI) |
| --- | --- | --- | --- | --- |
| Gender | Female n = 64 | Male n = 15 | n.s. |  |
| Age at surgery |  |  | n.s. |  |
| Stage |  |  | n.s. |  |
| Grade differentiation |  |  | n.s. |  |
| CD3 infiltration | High n = 42 | Low n = 36 | n.s |  |
| CD8 infiltration | High n = 24 | Low n = 55 | *p* = 0.003 | 0.28 (0.09 – 0.62) |
| CD4 infiltration | High n = 27 | Low n = 51 | n.s. |  |
| FoxP3 infiltration | Positive n = 57 | Negative n = 20 | n.s. |  |
| Adjuvancy | Yes n = 10 | No = 69 | n.s. |  |

n.s. = not statistically differences were found

**Supplementary Table 2: CD3^+^, CD8^+^ and Foxp3^+^ T cell infiltration related to patient’s demographic characteristics.**

| **Variable** | **Low**  **CD3^+^** | **High**  **CD3^+^** | **p value** | **Low**  **CD8^+^** | **High**  **CD8^+^** | **p value** | **Foxp3 (-)** | **Foxp3 (+)** | **p value** |
| --- | --- | --- | --- | --- | --- | --- | --- | --- | --- |
| **Gender** (n) |  |  | 0.19 § |  |  | 0.36 § |  |  | 0.42 § |
| Female | 32 | 31 |  | 42 | 22 |  | 18 | 45 |  |
| Male | 11 | 5 |  | 13 | 3 |  | 3 | 13 |  |
| **Age** (Mean; SEM) | 62.6 (2.3) | 61.4 (1.8) | 0.68 ¥ | 62.51 (1.6) | 62.68 (2.1) | 0.77 ¥ | 65.1 (2.5) | 61 (3.2) | 0.93 ¥ |
| **Stage (n)** |  |  | 0.53 ‡ |  |  | 0.38 ‡ |  |  | 0.39 ‡ |
| 0 | 2 | 2 |  | 4 | 0 |  | 1 | 3 |  |
| I | 10 | 6 |  | 10 | 5 |  | 7 | 9 |  |
| II | 7 | 2 |  | 7 | 2 |  | 2 | 7 |  |
| III | 22 | 22 |  | 29 | 16 |  | 8 | 36 |  |
| IV | 2 | 3 |  | 4 | 1 |  | 1 | 3 |  |
| **Histologic Grade** (n) |  |  | 0.30 ‡ |  |  | 0.55 ‡ |  |  | 0.90 ‡ |
| Well differentiated | 7 | 7 |  | 13 | 6 |  | 4 | 10 |  |
| Moderated | 24 | 15 |  | 30 | 9 |  | 10 | 29 |  |
| Undifferentiated | 11 | 15 |  | 12 | 8 |  | 8 | 18 |  |
| **Adjuvant Therapy** (n) |  |  | 0.63 § |  |  | 0.05 § |  |  | 0.16 § |
| Adjuvancy | 6 | 5 |  | 5 | 6 |  | 1 | 10 |  |
| No adjuvancy | 25 | 15 |  | 31 | 8 |  | 12 | 28 |  |
| * data not available from 29 patients |  |  |  |  |  |  |  |  |  |

§ Fisher’s exact test

¥ Unpaired t test

‡ Chi‑square test for trend

**Supplemental Figure 1: Overall survival of gallbladder cancer patients.** Kaplan-Meier survival curves for GBC patients stratified according to the disease stage.

**A**

**B**

**C**

**Supplemental Figure 2: CD4^+^ T cell infiltration in gallbladder cancer tissues does not correlates with patient survival.** Kaplan-Meier survival curves for patients with high and low CD4^+^ T cell infiltration in primary tumors based on Sturges’ Rule: **(A)** All stages; **(B)** Early stages (0, I); and **(C)** Late stages (II, IIIA, IIIB, and IV). The percentage of patients that lived at least 5 years after GBC diagnosis is depicted to the right of each curve.
